# Reclassification of the *HPGD* p.Ala13Glu variant causing primary hypertrophic osteoarthropathy

**DOI:** 10.1101/mcs.a006291

**Published:** 2023-12

**Authors:** Juan J. Alban, Alejandra Arango-Ramirez, Jorge A. Olave-Rodriguez, Jose A. Nastasi-Catanese, Lisa X. Rodriguez

**Affiliations:** 1 Fundación Valle del Lili, Center of Clinical Research, Cali, 760026, Colombia;; 2 Faculty of Health Sciences, Icesi University, Cali, 760031, Colombia;; 3 Fundación Valle del Lili, Department of Human Genetics, Cali, 760026, Colombia

**Keywords:** abnormality of the musculature of the limbs

## Abstract

Here, we highlight the case of a 31-yr-old man who had clinical features of primary hypertrophic osteoarthropathy (PHOAR) and harbored a homozygous variant (c.38C > A, p.Ala13Glu) in the *HPGD* gene, as indicated by whole-exome sequencing (WES). This variant has been previously classified by our laboratory as a variant of uncertain significance (VUS). However, another patient with the same phenotype and the same homozygous variant in *HPGD* was subsequently reported. In reassessing the variant, the absence of this variant in the gnomAD population database, supporting computational predictions, observation in homozygosity in two probands, and specificity of the phenotype for *HPGD*, all provide sufficient evidence to reclassify the *HPGD* c.38C > A, p.Ala13Glu variant as likely pathogenic.

## CASE PRESENTATION

**Figure 1.**
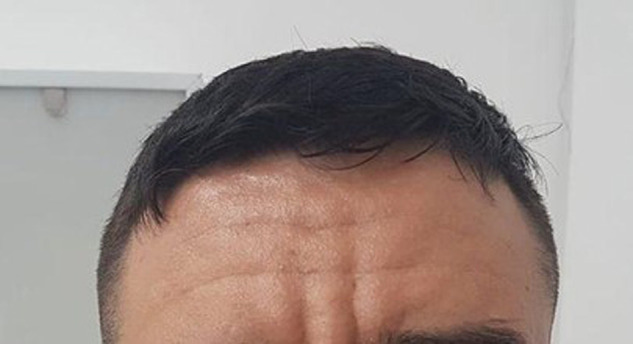
Coarse features and marked skin folds in the forehead, consistent with cutis verata gyrata.

**Figure 2.**
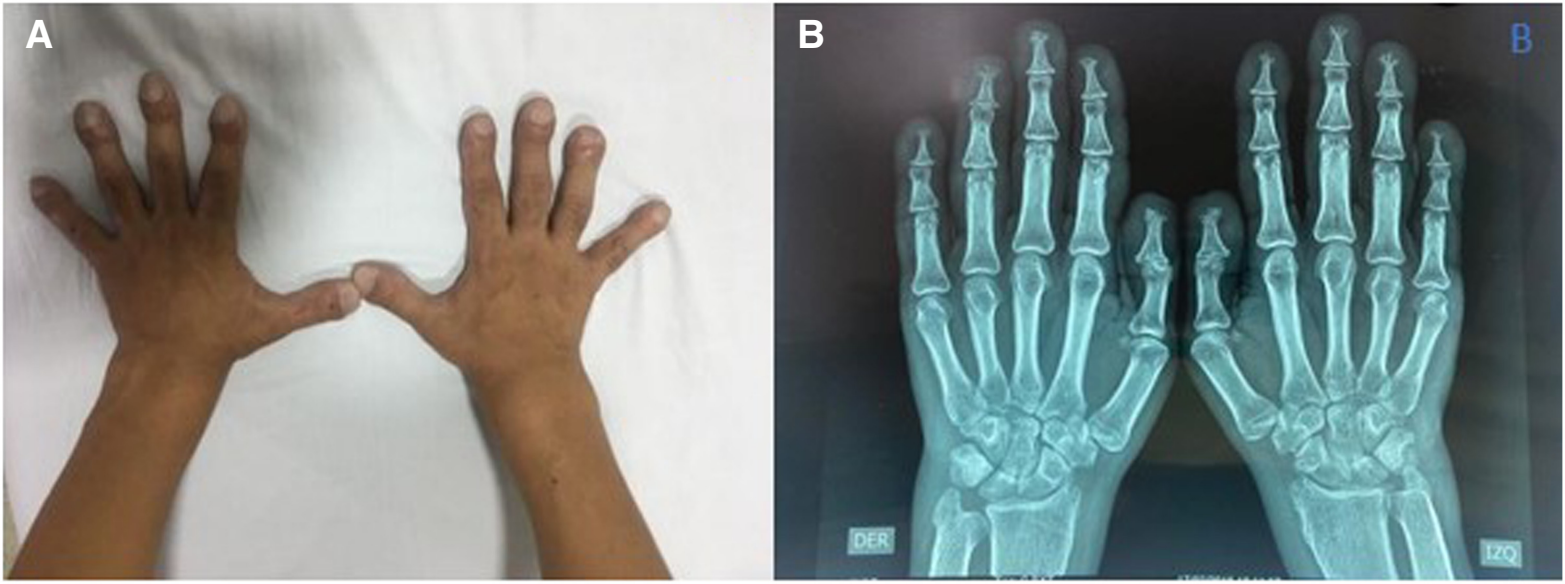
(*A*) Clubbed fingers with distal thumb deviation. (*B*) Anteroposterior X-ray of both hands show bone resorption of the distal phalanges (acro-osteolysis).

**Figure 3.**
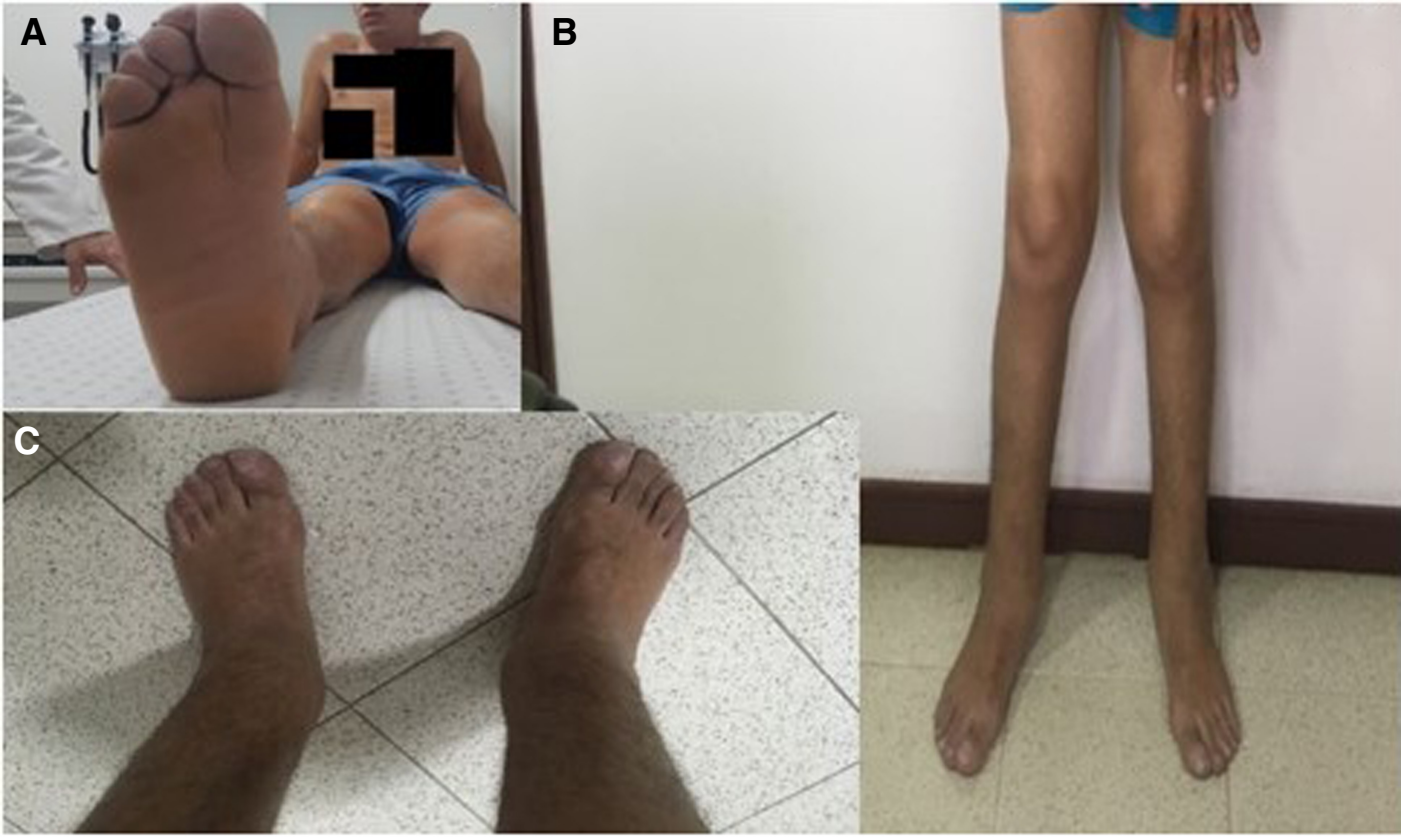
(*A*–*C*) Grade III edema alongside skin desquamation of the soles.

**Figure 4.**
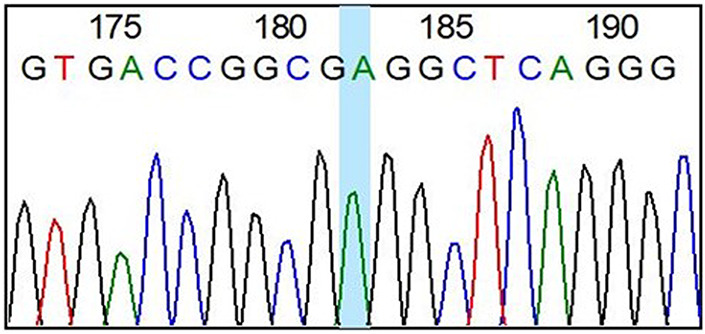
*HPGD* c.38C > A, p.Ala13Glu by Sanger sequencing.

We present a 31-yr-old male with the phenotype of primary hypertrophic osteoarthropathy (PHOAR1). The proband is the third child of healthy nonconsanguineous parents with no relevant family history. He was referred to our institution for overgrowing hands and feet and joint pain. The patient mentioned large hands and feet since birth, with further size exacerbation during puberty. On physical examination, his face exhibited coarse features and prominent facial folds in the forehead (Fig. 1). He had enlargement of the hands and feet, digital clubbing (Fig. 2A,B), deviation of both thumbs, pachydermia, hyperhidrosis, palmoplantar hyperkeratosis, arthralgia, arthritis, swollen joints, edema, and desquamation of the skin on the soles of the feet (Fig. 3A–C). Last year, he began complaining of emesis, nausea, fatigue, chronic gastritis, hypertrophic gastropathy, inflammatory bowel disease, considerable weight loss, and increased limb pain. The patient has been treated with mesalazine, hydrotherapy, anti-inflammatories, and analgesics with moderate response to treatment. Our patient carried a homozygous missense variant (NM_000860.6 c.38C > A, (p.Ala13Glu); Table 1) in the *HPGD* gene that was identified through whole-exome sequencing (WES) (Fig. 4).

**Table 1.** Variant interpretation of *HPGD*

Gene	Chromosome	HGVS DNA reference	HGVS protein reference	Variant type	Predicted effect	dbSNP/dbVar ID	Genotype	ClinVar ID
*HPGD*	4 4:174522414:G:T	NM_000860.6 c.38C > A	p.Ala13Glu	SNV	Substitution	No result	Homozygous	2058441

## VARIANT INTERPRETATION

This variant was initially classified as a variant of uncertain significance (VUS) (according to ACMG PP3 and PM2 pathogenic evidence criteria), which was consistent with a single submission to ClinVar (classified by Invitae as VUS on March 20, 2022; SCV004011732.1). To determine the inheritance, both mother and father, who were asymptomatic, were screened for the variant, and they were found to be heterozygous, confirming autosomal recessive inheritance. The variant is absent in the gnomAD population database (PM2_Supporting). Computational prediction tools (SIFT: “Deleterious”; PolyPhen-2: “Possibly Damaging”; Align-GVGD: “Class C0” described in SCV SCV004011732.1) support that it has a deleterious effect on the protein (PP3).

Another individual with the same phenotype (severe polyarthralgia, enlargement of the fingertips and toes, coarse facial features, prominent facial folds in the forehead, digital clubbing) who harbors the same homozygous variant in *HPGD* was subsequently reported (González et al. 2022), allowing application of PM3 given the now two homozygous individuals, per ClinGen guidance (https://clinicalgenome.org/site/assets/files/3717/svi_proposal_for_pm3_criterion_-_version_1.pdf). Additionally, PP4_Moderate was applied given that the phenotype is highly specific for the *HPGD* gene.

The *HPGD* gene is located on Chromosome 4 (Table 1) and encodes a 266-amino acid protein. This gene encodes a member of the short-chain nonmetalloenzyme alcohol dehydrogenase protein family. The encoded enzyme catalyzes the NAD-dependent oxidation of a broad range of hydroxylated polyunsaturated fatty acids (eicosanoids, docosanoids, including prostaglandins, lipoxins, and resolvins), producing their corresponding keto (oxo) metabolites. The enzyme reduces the levels of proproliferative prostaglandins, such as prostaglandin E2, and generates oxo-fatty acid products that can profoundly influence cell function by abolishing proinflammatory cytokine expression. It converts resolvins E1, D1, and D2 to their oxo products, which represents a mode of resolvin inactivation. Resolvin E1 plays an important role during the resolution phase of acute inflammation, whereas resolvins D1 and D2 have a unique role in obesity-induced adipose inflammation (Surhone et al. 2011; Stelzer et al. 2016). Pathogenic variants are located throughout the gene. In this case, the variant is located in the first turn of the protein and changes alanine to glutamic acid before the start of the protein helix. This change could alter the proper conformation around the substrate binding site, blocking or inhibiting the adequate union of the enzyme to the substrate. The clinical and physiological manifestations of PHOAR are related to the activity of prostaglandin E (Uppal et al. 2008; Sasaki et al. 2012). Not all patients, including the one presented here, manifest PHOAR from birth. In this case, our patient showed overgrowth of both hands and feet since puberty, and this was associated with joint pain. Additionally, prostaglandin E2 exhibits strong peripheral vasodilatory activity, which may explain finger clubbing and skin thickening due to prolonged local vasodilation (Uppal et al. 2008). The patient presented with skin thickening, palmoplantar hyperkeratosis, and hyperhidrosis. The clinical signs and symptoms of the disease tend to stabilize over time, and there is no specific treatment. However, there are therapeutic options for the control of symptoms with aspirin, nonsteroidal anti-inflammatory drugs (NSAIDs), systemic corticosteroids, and colchicine. Plastic surgery is reserved for patients with significant eyelid ptosis or severe aesthetic problems (Salah et al. 2019). In this case, controlling the patient's joint pain was difficult using aspirin and NSAIDs. Currently, he has no intention of undergoing plastic surgery.

Inheritance patterns in PHOAR vary according to the study. Initially, PHOAR genes were first found in consanguineous families, suggesting autosomal recessive inheritance. Nonetheless, other studies have supported autosomal dominant transmission with incomplete penetrance (Castori et al. 2005). Similarly, its expression varies by sex, with a higher tendency in males, and presents an early puberty onset. However, in this patient, the symptoms manifested during early adulthood. PHOAR 2 tends to have a more skewed sex ratio than PHOAR 1. Some studies have demonstrated that prostaglandin transport is influenced by sex hormones.

Regarding the current patient, gastrointestinal alterations have been reported in PHOAR. Castori et al. (2005) noted that gastrointestinal involvement varies depending on the inheritance pattern of the condition, including chronic gastritis, hypertrophic gastropathy, peptic ulcer, and Crohn's disease. The overall prevalence of gastrointestinal signs was 10%–12%. Although Crohn's disease and peptic ulcers were observed in both dominant and recessive forms, chronic/hypertrophic gastritis is more common in the autosomal dominant form.

## SUMMARY

This case involves a homozygous missense variant p.Ala13Glu that demonstrates autosomal recessive inheritance. Biallelic truncating and missense variants have been identified to affect unrelated families with PHOAR. Mild digital clubbing is observed in heterozygous family members, particularly older individuals. Out of 41 published variants, 16 (39%) were missense, 12 (29%) were frameshift, 2 (5%) were noncoding, 0 (0%) were synonymous, 1 (2.5%) was nonsense, 7 (17%) were splice junction loss, and 3 (7.5%) were start loss. Among these 41 mutations, 25 were classified as pathogenic and 16 were classified as likely pathogenic according to American College of Medical Genetics and Genomics (ACMG) criteria (Kopanos et al. 2019).

In Colombia, only two cases of PHO have been published. One case was a clinical diagnosis in a 17-yr-old patient (Moreno et al. 2010), and the other case, as described above, was a molecular diagnosis with the variant classified as a VUS (González et al. 2022). Furthermore, our patient presented clinical features of PHO and carried the same variant that was previously reported in ClinVar as a VUS. The absence of this variant in the gnomAD population database, computational predictions, observation in homozygosity in two probands, and specificity of the phenotype for *HPGD* provide sufficient evidence to reclassify the *HPGD* c.38C > A, p.Ala13Glu variant as likely pathogenic.

In conclusion, we describe the reclassification of a variant in the *HPGD* gene that is associated with primary hypertrophic osteoarthropathy. To our knowledge, this is the second Colombian patient with molecular confirmation. Clinical evidence of overgrowth, arthralgia, skin thickening, and gastrointestinal manifestations should raise suspicion of PHOAR and prompt genetic testing. Further research is required to explore prognosis, new therapeutic interventions, and functional studies of the variant to demonstrate pathogenicity in vitro.

## ADDITIONAL INFORMATION

### Data Deposition and Access:

The variant of this patient was submitted to ClinVar (https://www.ncbi.nlm.nih.gov/clinvar/) and can be found under accession number SCV004011732.1.

### Ethics Statement

Informed consent was signed by the patient, and the study was approved by the ethical committee of the Fundación Valle del Lili.

### Acknowledgments

We thank the patient and his family for their generosity in sharing clinical data and laboratories for the ongoing data sharing in ClinVar.

### Author Contributions

L.X.R. and J.J.A. wrote the manuscript; J.A.N.-C. and L.X.R. reclassified the variant; and A.A.-R. and J.A.O.-R. edited the manuscript.

### Funding

This study was supported in part by Fundación Valle del Lili in conjunction with the Faculty of Health Sciences, Icesi University. The content is solely the responsibility of the authors.

### Competing Interest Statement

The authors have declared no competing interest.
